# SCOPe: improvements to the structural classification of proteins – extended database to facilitate variant interpretation and machine learning

**DOI:** 10.1093/nar/gkab1054

**Published:** 2021-12-01

**Authors:** John-Marc Chandonia, Lindsey Guan, Shiangyi Lin, Changhua Yu, Naomi K Fox, Steven E Brenner

**Affiliations:** Environmental Genomics and Systems Biology Division, Lawrence Berkeley National Laboratory, Berkeley, CA 94720, USA; Molecular Biophysics and Integrated Bioimaging Division, Lawrence Berkeley National Laboratory, Berkeley, CA 94720, USA; Department of Plant and Microbial Biology, University of California, Berkeley, CA 94720, USA; Department of Plant and Microbial Biology, University of California, Berkeley, CA 94720, USA; College of Engineering, University of California, Berkeley, CA 94720, USA; Department of Plant and Microbial Biology, University of California, Berkeley, CA 94720, USA; Environmental Genomics and Systems Biology Division, Lawrence Berkeley National Laboratory, Berkeley, CA 94720, USA; Molecular Biophysics and Integrated Bioimaging Division, Lawrence Berkeley National Laboratory, Berkeley, CA 94720, USA; Environmental Genomics and Systems Biology Division, Lawrence Berkeley National Laboratory, Berkeley, CA 94720, USA; Department of Plant and Microbial Biology, University of California, Berkeley, CA 94720, USA; College of Engineering, University of California, Berkeley, CA 94720, USA

## Abstract

The Structural Classification of Proteins—extended (SCOPe, https://scop.berkeley.edu) knowledgebase aims to provide an accurate, detailed, and comprehensive description of the structural and evolutionary relationships amongst the majority of proteins of known structure, along with resources for analyzing the protein structures and their sequences. Structures from the PDB are divided into domains and classified using a combination of manual curation and highly precise automated methods. In the current release of SCOPe, 2.08, we have developed search and display tools for analysis of genetic variants we mapped to structures classified in SCOPe. In order to improve the utility of SCOPe to automated methods such as deep learning classifiers that rely on multiple alignment of sequences of homologous proteins, we have introduced new machine-parseable annotations that indicate aberrant structures as well as domains that are distinguished by a smaller repeat unit. We also classified structures from 74 of the largest Pfam families not previously classified in SCOPe, and we improved our algorithm to remove N- and C-terminal cloning, expression and purification sequences from SCOPe domains. SCOPe 2.08-stable classifies 106 976 PDB entries (about 60% of PDB entries).

## INTRODUCTION

Nearly all proteins have structural similarities with other proteins, and in some of these cases, share a common evolutionary origin. First released to the public 27 years ago, the Structural Classification of Proteins (SCOP) database (1–4) was a manually curated hierarchy of domains from all proteins of known structure, organized according to their structural and evolutionary relationships. Work on the classic SCOP database concluded in 2009 with the release of SCOP 1.75. Since that time, we have maintained the successor knowledgebase SCOPe (SCOP–extended) (5–7) in order to provide ongoing updates to the hierarchy and classification of new protein structures from the Protein Data Bank (PDB) (8,9). Comparisons of SCOPe to other structural classifications of proteins including SCOP2 (10), CATH (11) and ECOD (12), as well as to the sequence-based Pfam classification (13) are provided elsewhere (6,7,14).

Like SCOP, the SCOPe knowledgebase aims to provide authoritative information about proteins’ evolutionary relatives, particularly those too ancient to be readily recognized from sequence. SCOPe organizes protein domains into a hierarchy that includes the following levels: a *Family* contains related proteins with similar sequences but typically distinct functions. The *Superfamily* level brings together protein families with common functional and structural features inferred to share a common ancestor. Near the root, the basis of classification is purely structural: similar superfamilies without compelling evidence of a common evolutionary origin are grouped into *Folds*, which are arranged into *Classes* based mainly on secondary structure content and organization (15). Classification at the *Superfamily* level in particular depends on expert curation to integrate many types of information. To identify ancient homologous relationships, proteins with similar three-dimensional structure and no recognizable sequence similarity are examined to determine whether they possess structural and functional features indicative of homology. If convincing evidence is found of an evolutionary relationship, this is annotated by grouping the homologous domains into a single *Superfamily*; otherwise, the domains of similar structure are annotated as having a common *Fold* but not grouped into a *Superfamily*.

In addition to classifying domains, SCOPe includes a number of additional resources to support computational analyses of the protein structures and their evolutionary relationships. We provide sequences and PDB-style coordinate files for all SCOPe domains, as well as sequences for all PDB chains that are classified in SCOPe. Post-translationally modified amino acids are translated back to the original sequence, and sequences are curated to eliminate any errors resulting from automated parsing of PDB files. Because the majority of sequences in the PDB have high similarity to others, SCOPe provides representative subsets of proteins that span all classified protein structures or domains; this alleviates bias towards proteins experimentally characterized many times. The highest quality representative in each subset is chosen using AEROSPACI scores (16), which provide a numeric estimate of the quality and precision of crystal structures. All data may be downloaded in parseable files, or in a SQL database. All data are also archived off-site and available for download from Zenodo, an open-access data repository.

Since long before the FAIR principles were formalized (17), we have attempted to ensure that all SCOPe data be Findable, Accessible, Interoperable and Reusable, both for machines and for people. To enable findability, both SCOP and SCOPe have been made available as versioned releases, annually on average, since their inception, and the SCOPe website provides interactive access to all SCOP and SCOPe releases published since 2001. We publish two kinds of SCOPe releases: major ‘stable’ releases that contain updates to the hierarchic structure, manual curation, and error correction relative to previous stable releases; and minor periodic updates (7). Once each stable release is published, no changes or corrections may be made until the next stable release. However, in an effort to stay more closely synchronized with the PDB, we supplement the stable releases with periodic updates (approximately monthly). Hundreds of additional entries are added to SCOPe each month, because after at least one structure from a SCOPe family has been classified by a human curator, most other structures from that family may be added automatically by our rigorously validated software pipeline (5). These updates are versioned by combining the stable release number with the update date (e.g., ‘2.07–2021-07–07’). All versions of both SCOP and SCOPe released since 2001 have featured ‘stable identifiers’ for each data object, meaning that the same identifiers always refer to the same data objects between releases. If changes are made to the underlying data object, the identifier is retired and replaced with a new one. To ensure interoperability and reusability, SCOPe uses the same identifiers, assigned using the same well-defined rules, as the classic SCOP releases.

The current SCOPe release, 2.08, classifies 344 851 domains from 106 976 PDB entries; the previous stable SCOPe release, 2.07, classifies 276 231 domains from 87 224 PDB entries, and SCOP 1.75 classifies 110 800 domains from 38 221 PDB entries. New features in SCOPe 2.08 are described below.

## VARIANT INTERPRETATION

The cost of sequencing a human genome has now dipped below $1000, so hundreds of thousands are expected to be sequenced each year. We therefore have developed search and display tools to aid in the interpretation of the phenotypic impact of variants identified in individuals’ genomes. Genome variant analysis often first involves rejecting the vast majority of variants that are unlikely to have a deleterious or interesting phenotype. When variants affect a coding region, structural data about the protein or its homologs may be particularly valuable (18,19). For example, a study by our colleagues involved a structural analysis of the replacement of arginine with a tryptophan residue (R192W) in the ZAP-70 protein's C-terminal. The analysis predicted that this sequence change has a structural impact on a phosphotyrosine-binding pocket and may diminish ZAP-70 binding to the ζ-chain, contributing to a novel human autoimmune disease (19).

To assist in the analysis of genetic variants and to enable easier access to structural classification data, we built a search tool to map human genetic variants to protein structures and associated SCOPe data. This tool works for both novel and previously reported variants. Users can search for structures relevant to a variant of interest by providing HGVS expressions or genome coordinates using hg19 and GRCh38. HGVS expressions are a nomenclature developed with the Human Genome Variation Society, which describes the position of each variant relative to a locus or reference sequence. We use Ensembl's Variant Effect Predictor (VEP) tool (20) to retrieve associated Ensembl reference transcripts, gene name, predicted variant consequence, and affected protein position of the variant provided by the user. Transcripts and gene names are mapped to UniProt canonical proteins using SwissProt (21) and variant positions in the UniProt canonical protein are mapped and translated to a residue-level annotation of structures in the PDB using SIFTS (Structure Integration with Function, Taxonomy and Sequence) (22). Finally, PDB SEQRES indices identified using SIFTS are mapped to their corresponding positions in the PDB ATOM records using RAF (Rapid Access Format) annotations (16) that are part of SCOPe.

**Figure 1.**
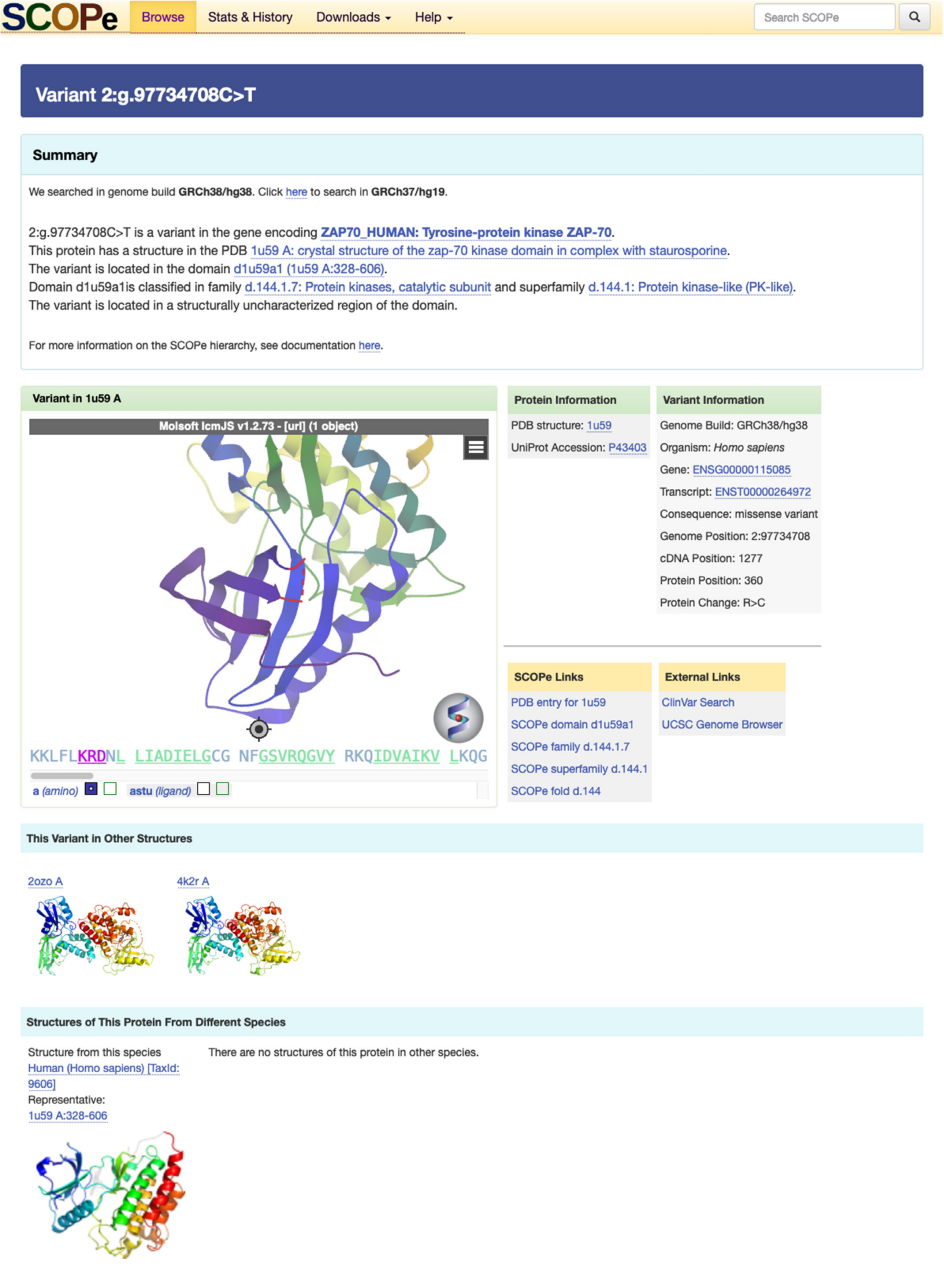
Variant search results. The variant search result page displays an interactive viewer showing the structural context of the variant and relevant evolutionary context from the SCOPe hierarchy, including members of the same family or superfamily as the impacted protein domain. In the example shown, the user searched for a missense variant in chromosome 2, which affects the coding sequence of the ZAP-70 protein. The variant viewer displays the most relevant human ZAP-70 structure classified in SCOPe. Note that in this structure, the amino acid residue affected by the variant is located in a structurally uncharacterized loop in the protein, so the nearest residues in the structure are highlighted. Several additional ZAP-70 structures are also shown, allowing users to visualize the impact of the variant in different structural contexts.

In cases in which the variant is in a protein coding region, we map the location of the variant onto the structure, displayed to the user using the MolSoft molecule viewer (https://www.molsoft.com/). If the variant is in a structurally uncharacterized region of the protein, we report the nearest neighboring residues in the structure. Our viewer also displays relevant structural and evolutionary context from the SCOPe hierarchy, including members of the same family or superfamily as the impacted protein domain. Although our tools are currently limited to searching for and visualizing variant impacts in structurally characterized human proteins, future development will allow us to visualize the predicted impact of human variants using these homologous protein structures as well. See Figure 1 for an example variant from (19). For cases in which the variant is not in a protein coding region, we report to the user relevant genomic loci and a description of the predicted variant consequence.

### Annotation of structurally heterogeneous families

We have improved consistency in how structures in the same family are divided into domains, so that automated methods (e.g. deep learning classifiers) that rely on multiple alignments of homologous SCOPe domains will be less likely to produce incorrect results due to variable domain lengths within the same family. Early releases of SCOP were meant to be browsed by humans, and focused on correct identification of relationships rather than utility for algorithms; for example, gene duplication was often annotated in comments rather than in a machine-parseable format. As a result of this legacy, in some SCOPe families that arose from a gene duplication, some of the structures are split into two domains and others left as one. We have identified other types of structural heterogeneity as well, as detailed below. In many cases, we changed the outliers to be consistent with the most common structure in the family. All remaining cases we identified were annotated in our database. These annotations are displayed in our website and provided to the public through a new machine-parseable file (dir.inc; described below).

To locate potentially structurally heterogeneous domains, we assumed that if all domains in a family were structurally similar, the length of the domains would also be very similar. Thus, we collected domain length data and flagged families with significant length variation for manual review. Two different domain lengths were calculated: one based on PDB SEQRES records, the genetically encoded protein sequence; and one based on ATOM records, which were the parts of the protein that were experimentally observed (23). For large families, we detected length variation by applying kernel density estimation to the SEQRES lengths and counting and comparing the resulting peaks. For smaller families, we deployed a simpler method by calculating the ratio of the longest to shortest SEQRES length in the family. In addition, we also flagged domains and families if the SEQRES and ATOM sequence lengths were significantly different, or which contained human-readable metadata (clade names or comments) that suggested potential heterogeneity. We have also incorporated a version of this flagging system into our code for adding new, manually classified entries, in order to provide additional protection against manual errors such as typos.

**Figure 2.**
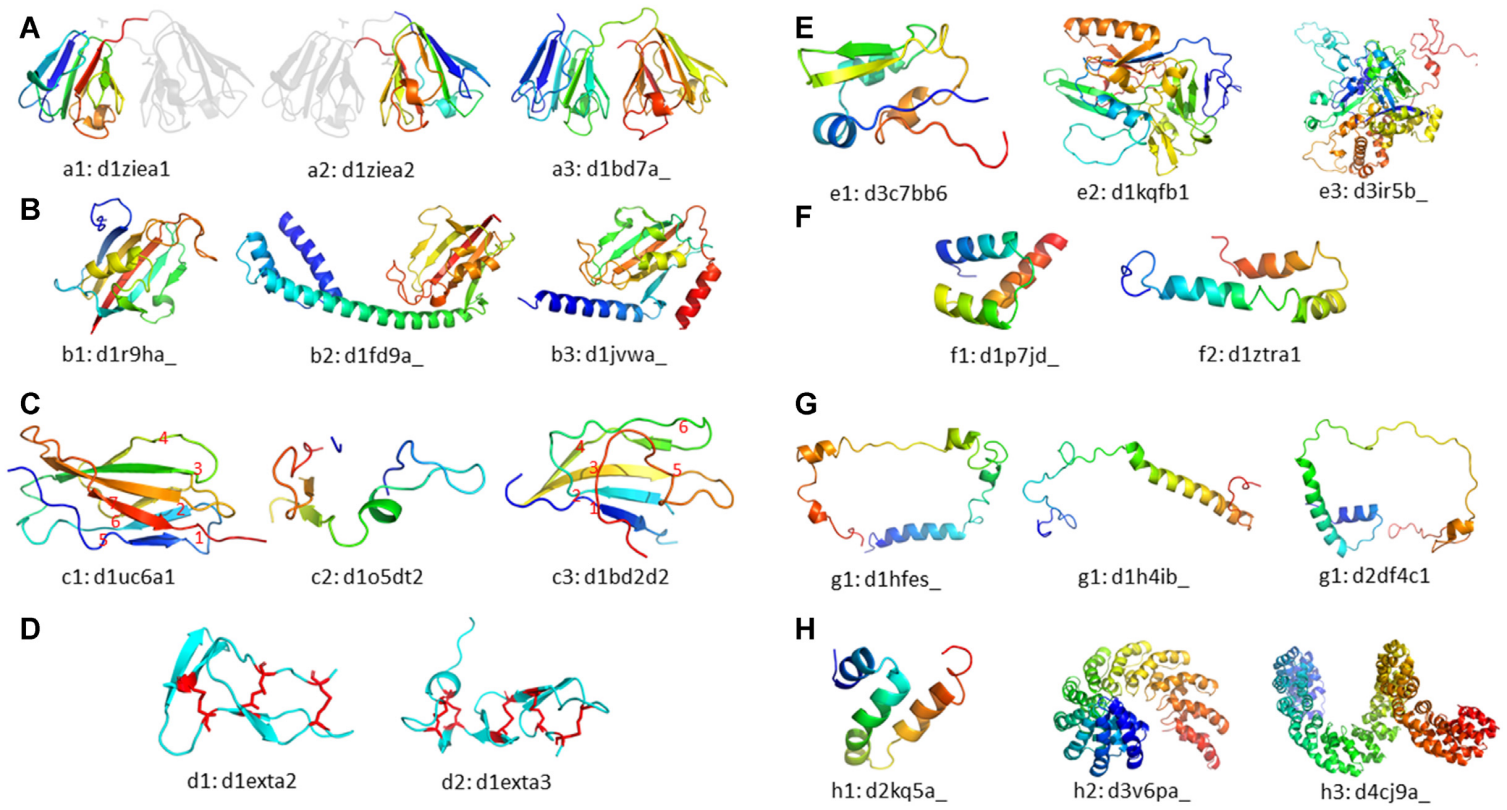
Examples of structurally heterogeneous families. (**A**) The three domains belong to family b.11.1.1: Crystallins/Ca-binding development proteins. Most entries in this family are divided into two 8-beta-strand domains, but a3 remains undivided. In this example, we label a3 as having multiple alternative domain divisions. (**B**) The three domains belong to family d.26.1.1: FKBP immunophilin/proline isomerase. Shown in b1 is the common family domain; shown in b2 is the common family domain followed by an N-terminal all-alpha subdomain; shown in b3 is the common family domain, with additional alpha-helices at both termini. (**C**) The three domains belong to family b.1.2.1: Fibronectin type III. Shown in c1 is the family-specific domain with 7 beta strands in 2 sheets; shown in c2 is a fragment which is missing more than one third of the beta strands presented in the common fold; shown in c3 is a domain missing one beta strand from the common fold. (**D**) The two domains belong to family g.24.1.1: TNF receptor-like, which is defined by a specific pattern of disulfide bonds. We have shown disulfide bonds as red sticks and observed there are different numbers of these bonds in each domain. Domains with more disulfide bonds (such as d2) will be labeled as having additional elements; likewise, domains with fewer disulfide bonds will be labeled as missing some elements. (**E**) The three domains belong to family d.58.1.5: Ferredoxin domains from multidomain proteins. As the family name suggests, we can see that domains in this family may contain different numbers of (sub)domains. (**F**) The two domains belong to family a.4.1.1: Homeodomain. Shown in f1 is the common domain, which has three alpha helices arranged into a triangle-like structure; shown in f2 is a domain with similar secondary structures and sequence as f1 but folded differently. (**G**) The three domains belong to different families under the same fold a.137: Non-globular all-alpha subunits of globular proteins, which is deemed ‘not a true fold’ in the comments by SCOP(e) curators. We include this label as a category here in order to make it accessible to automated methods. (**H**) The three domains belong to family a.298.1.1: TAL (transcription activator-like) effector. Shown in h1 is a single repeat unit; h2 and h3 contain different numbers of this same repeat unit.

After identifying potential heterogeneous families, we reviewed these families manually and identified 340 families and 6 folds total that contain structurally heterogeneous domains. We then annotated each entry with one or more of the following labels to describe the type of heterogeneity:

1. Multiple alternative domain divisions: Applies to families that are inconsistently divided into domains. See Figure 2A.
2. Additional (sub)domain(s): Applies to domains that contain additional (sub)domain(s) which are not in the common domain. See Figure 2B.
3. Additional insertion(s)/extension(s): Applies to domains that contain additional insertion(s) or extension(s) that are not arranged into (sub)domain(s). These can be additional secondary structures, significantly longer family-specific secondary structures, disordered loops, etc. See Figure 2B.
4. Fragment: Applies to domains that are missing at least 1/3 of the common fold in the family (based on missing either sequence or structure). See Figure 2C.
5. Missing some secondary structure(s): Applies to domains that are missing secondary structures from the family-specific domain (but still contain at least 2/3 of the structure, or these would be considered fragments). See Figure 2C.
6. Additional element(s): Applies to domains that contain additional secondary structure elements or disulfide bonds. See Figure 2D.
7. Missing element(s): Applies to domains that are missing some secondary structure elements or disulfide bonds. See Figure 2D.
8. Different number of (sub)domain(s) of a multi-domain family: Applies to all members of a family in SCOPe class e (multi-domain proteins) in which individual members contain different numbers of domain(s). See Figure 2E.
9. Heterogeneous fold: Applies to domains that adopt a different fold from the most common structure found in the fold, but which have similar sequence and number of secondary structure elements. This category does not include domain swaps (24). See Figure 2F.
10. Not a true fold: Applies to all domains of a fold that was described by SCOP(e) curators as ‘not a true fold.’ See Figure 2G.
11. Repeat and inconsistent: Applies to all domains of a family if the common domain is composed of a different number of small repeating units. See Figure 2H.

Users can download the parseable files from the ‘downloads’ section of our website, and find more documentation about the parseable file format in the ‘help’ section.

This method is still not comprehensive. For example, it cannot detect a heterogeneous family in which all domains have similar lengths; it also does poorly in detecting domains with small additional or missing structures. However, it is effective in flagging major differences such as large insertions or deletions. We plan to improve our method by including structural comparison to help identify more heterogeneity, especially for heterogeneous folds.

### Annotation of tandem repeat units

Some protein domains in SCOPe consist of a number of smaller tandem repeating units. The number of repeats may or may not be the same between the domains in the same family. To facilitate automated algorithms developed or trained on the SCOPe database, we provide machine-parseable annotations of the extent of a single repeat unit for all families of repeats in classes a to g. Tandem repeats are often also annotated in other databases, such as Pfam (13).

### Annotation of cloning, expression, and purification artifacts

Previously, in SCOPe version 2.06, we moved non-natural sequences that represented cloning, expression, or purification tags to a new class (l: Artifacts) in order to separate them from the homology-based curations and prevent spurious similarity between non-homologous protein sequences. Tags were identified using PDB metadata (SEQADV records) referring to cloning, expression, or purification tags at the N- or C-terminal of each chain, as described in more detail elsewhere (6).

In order to identify additional tags not annotated in PDB metadata in SCOPe version 2.08, we introduce an automated tag identification method using chain sequence comparisons to canonical UniProt proteins. For every chain annotated as belonging to a UniProt protein according to PDB DBREF records, the N- or C-terminal sequence is considered a putative tag if the tag sequence does not match the corresponding sequence in the UniProt protein and is less than 10 residues long. Additionally, the non-tag sequence of the chain's SEQRES sequence is compared to the corresponding UniProt protein sequence, to ensure the DBREF record is consistent with the expected sequence. In addition to the 32 871 tags we had previously annotated, in SCOPe 2.08 we annotated 2399 new tags using PDB metadata and 400 new tags using sequence comparisons to UniProt proteins.

## MANUAL CURATION PRIORITIES

As previously (6,7), we prioritized manual curation of new structures by focusing on those Pfam (13) families with the largest number of structures, but without any structure classified in SCOPe. This prioritization reflects the hypothesis that protein families classified in Pfam are likely to be of more interest to the biological community than proteins not in Pfam, as Pfam is human curated. We prioritized unclassified Pfam sequence families with the most three-dimensional structures characterized, because larger numbers of structures may reflect a greater degree of scientific interest, and because once one structure is manually classified, it may be used as a model for classifying other structures in the family. In the future, we will also consider classifying high quality predicted structures (25,26).

In SCOPe 2.08, we curated structures from the 74 largest Pfam families not classified previously, including all families with 25 or more structures. As we previously found, the relationship between Pfam families and SCOPe families (or superfamilies) is not 1:1. Among the 74 Pfam families, 22 (30%) had at least one domain classified into a new SCOPe fold, 10 (14%) into a new superfamily in an existing fold, 33 (45%) into a new family within an existing superfamily, and 9 (12%) as new proteins within an existing family. These results are similar to the novelty of newly classified structures in SCOP and SCOPe, as we previously reported (6).

### New parseable files and RAF format

Annotations of structural heterogeneity and repeat units are provided in new machine-parseable files, similar to the dir.* files released with previous versions of SCOP and SCOPe (2). The dir.inc.scope.txt file, which annotates structural inconsistencies contains three tab-delimited columns: the sccs identifier for the structurally heterogeneous clade, a two-letter code indicating the type of heterogeneity, and a list of sid identifiers for the inconsistent domains in the clade. The dir.rep.scope.txt file, which annotates repeats, contains three tab-delimited columns: the sccs identifier for the family defined by a repeat, the sid of the domain in which the canonical repeat is defined, and the beginning and end of a single repeat unit inside that domain.

We have also made a minor update to the RAF maps, which summarize the SEQRES - ATOM relationship within each protein chain in a form that can be rapidly parsed in most computer languages (27). Residues present in the SEQRES but missing from the ATOM records are now assigned residue identifiers by the PDB and documented in the mmCIF and XML format files. We now include these identifiers in the RAF format (version 0.03) instead of the letters (‘B’, ‘M’ and ‘E’) that we previously used to indicate missing identifiers.

## Notes

Present address: Changhua Yu, Division of Biology and Biological Engineering, California Institute of Technology, 1200 East California Boulevard, Pasedena, CA 91125, USA.

Present address: Naomi K. Fox, Invitae; 458 Brannan St, San Francisco, CA 94107, USA.
